# High-Efficacy α,β-Dehydromonacolin S Improves Hepatic Steatosis and Suppresses Gluconeogenesis Pathway in High-Fat Diet-Induced Obese Rats

**DOI:** 10.3390/ph14040375

**Published:** 2021-04-17

**Authors:** Jutatip Kaewmalee, Atcharaporn Ontawong, Acharaporn Duangjai, Chittreeya Tansakul, Vatcharin Rukachaisirikul, Chatchai Muanprasat, Chutima Srimaroeng

**Affiliations:** 1 Department of Physiology, Faculty of Medicine, Chiang Mai University, Chiang Mai 50200, Thailand; jutatip.kml@gmail.com; 2 Division of Physiology, School of Medical Sciences, University of Phayao, Phayao 56000, Thailand; atcharaporn.on@up.ac.th (A.O.); achara.phso@gmail.com (A.D.); 3 Division of Physical Science and Center of Excellence for Innovation in Chemistry, Faculty of Science, Prince of Songkla University, Hat Yai, Songkhla 90112, Thailand; chittreeya.t@psu.ac.th (C.T.); vatcharin.r@psu.ac.th (V.R.); 4 Chakri Naruebodindra Medical Institute, Faculty of Medicine Ramathibodi Hospital, Mahidol University, Samutprakarn 10540, Thailand; chatchai.mua@mahidol.ac.th

**Keywords:** statin, α,β-dehydromonacolin S, lovastatin analog, 3-hydroxy-3-methylglutaryl coenzyme A reductase inhibitor, hepatic steatosis, type 2 diabetes mellitus, gluconeogenesis

## Abstract

Isolated α,β-dehydromonacolin S (*C5*) from soil-derived fungus *Aspergillus sclerotiorum* PSU-RSPG178 was recently shown to exhibit an inhibitory effect against 3-hydroxy-3-methylglutaryl coenzyme A reductase (HMGR) activity in vitro. In this study, we investigated the effects of *C5* on lipid-lowering, hepatic steatosis, and hepatic gluconeogenesis in vivo. The control rats received a daily dose of either vehicle or *C5* at 10 mg/kg, while the high-fat diet-induced obese (HFD) rats were administered vehicle; 1, 3, or 10 mg/kg *C5*; or 10 mg/kg lovastatin (LO) for 6 weeks. *C5* significantly improved dyslipidemia and diminished liver enzymes, HMGR activity, insulin resistance, and hepatic steatosis, comparable to LO without any hepatotoxicity and nephrotoxicity in HFD rats. A higher efficacy of *C5* in lipid-lowering activity and anti-hepatic steatosis was associated with a significant decrease in genes involved in lipid metabolism including sterol regulatory element binding protein (SREBP) 1c, SREBP2, liver X receptor alpha (LXRα), and peroxisome proliferator-activated receptor (PPAR) gamma (PPARγ) together with an increase in the PPAR alpha (PPARα). Correspondingly, *C5* was able to down-regulate the lipid transporters cluster of differentiation 36 (CD36) and Niemann-Pick C1 Like 1 (NPC1L1), increase the antioxidant superoxide dismutase gene expression, and decrease the proinflammatory cytokines, tumor necrosis factor alpha (TNFα) and interleukin 1 beta (IL-1β). Impairment of hepatic gluconeogenesis and insulin resistance in HFD rats was restored by *C5* through down-regulation of the gluconeogenic genes phosphoenolpyruvate carboxykinase (PEPCK) and glucose-6-phosphatase (G6Pase), and the activation of AMP-dependent kinase serine (AMPK) and serine/threonine protein kinase B (Akt). Collectively, this novel *C5* may be a therapeutic option for treating dyslipidemia, hepatic steatosis, and reducing potential risk for diabetes mellitus.

## 1. Introduction

An imbalance between a high-fat diet (HFD) and internal de novo synthesis was shown to be the primary factor contributing to lipid dyshomeostasis as a consequence of hepatic lipid accumulation and non-alcoholic fatty liver disease (NAFLD), which could develop from simple steatosis to nonalcoholic steatohepatitis (NASH), advanced fibrosis, and cirrhosis [1]. Several studies demonstrated that HFD-feeding in rodents can induce oxidative stress and cholesterol biosynthesis and increase hepatic triglycerides and total cholesterol contents, leading to hepatic lipid accumulation [2,3]. In addition, a previous study found that sterol regulatory element-binding protein 2 (SREBP2) was upregulated after HFD consumption, resulting in the increased expression of 3-hydroxy-3-methylglutaryl coenzyme A reductase (HMGR), the rate-limiting enzyme of de novo hepatic cholesterol synthesis, in a mouse model [4]. A recent study indicated that abnormal insulin-induced gene 1 expression, a key suppressor of SREBP activation and an accelerator of HMGR degradation, was involved with NAFLD and diabetic dyslipidemia in both in vitro and in vivo, as well as in obese patients [5]. The activation of liver X receptor (LXR) by oxysterol led to enhanced fatty acid synthesis and hepatic lipid synthesis in both in vitro and in vivo [6]. Thus, modulation of hepatic lipid content, predominantly through key regulators involved in lipid metabolism and transporters, is thought to be the primary target for the prevention of lipid dyshomeostasis and its hepatic complications. 

At present, there are several lipid-lowering drugs, such as statins, fibrates, bile acid sequestrants, cholesterol absorption inhibitors, and novel inhibitors of PCSK9-mediated low-density lipoprotein receptor (LDLR) degradation. Among these, statins are the most widely prescribed class of drugs for lowering total cholesterol and low-density lipoprotein cholesterol (LDL), as well as lowering triglycerides and elevation of high-density lipoprotein cholesterol (HDL) [7]. Statins are either derived from micro-organisms through biotechnology (fermentation-derived statins (type 1)) or from chemical synthesis (type 2), and statins can also be classified on the basis of their solubility properties [8]. For instance, atorvastatin, simvastatin, lovastatin, fluvastatin, cerivastatin, and pitavastatin are lipophilic pro-drugs that require the phase II hepatic metabolizing enzyme cytochrome C P450, while pravastatin and rosuvastatin are hydrophilic and readily active agents [9]. Statins appear to have numerous additional effects, e.g., the promotion of the proliferation of new blood vessels, the oxidative modification of LDL, and antioxidant and anti-inflammatory properties [10]. Nonetheless, adverse effects of statins have been reported. Pravastatin and atorvastatin were shown to upregulate the expression of hepatic gluconeogenic genes, including phosphoenolpyruvate carboxykinase (PEPCK) and glucose-6-phosphatase (G6Pase). They also impaired oral glucose tolerance in mice [11]. Similarly, simvastatin acutely increased hepatic gluconeogenesis and blood glucose level in mice [12]. Correspondingly, long-term treatment of statins is associated with a risk of a new-onset of type 2 diabetes (NOD) in humans [13].

Lovastatin (LO) is the first statin to be developed that has been shown to effectively reduce LDL in healthy volunteers [14]. In addition to inhibiting HMGR activity, LO exhibits antioxidant properties by increasing the activity of catalase and glutathione peroxidase enzymes in H_2_O_2_-induced oxidative stress in rats [15]. The anti-inflammatory effect of LO was also demonstrated by suppressing the production of nitric oxide (NO), tumor necrosis factor alpha (TNFα), inducible NO synthase (iNOS), and nuclear factor kappa B (NF-κB) in RAW264.7 macrophage cells [16]. In addition, lovastatin was reproposed as a breast cancer agent [17]. More recently, combination of lovastatin with either metformin or gliclazide had higher efficacy on hepatoprotection than traditional anti-diabetic drug in diabetic mice [18].

**Figure 1:**
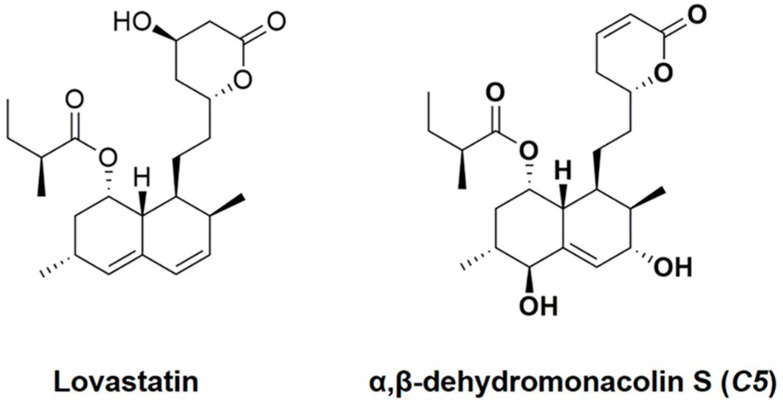
Structures of lovastatin (LO) and α,β-dehydromonacolin S (*C5*).

From the milestone of development of lipid-lowering agents, the exploration of efficient active compounds from natural sources, including metabolites from soil fungi, is a continuing challenge. Our previous study found that α,β-dehydromonacolin S (*C5*) is a novel lovastatin analog produced by the soil-derived fungus *Aspergillus sclerotiorum* PSU-RSPG178. Due to *C5*’s chemical structure (Figure 1), it exhibits high potency against HMGR activity in vitro [19]. However, the effects of *C5* on lipid-lowering, hepatic steatosis, and hepatic gluconeogenesis in vivo has not yet been explored. Accordingly, in this study, we investigated the effect of the *C5* on lipid homeostasis, insulin resistance, hepatic steatosis, and hepatic gluconeogenesis in high-fat diet-induced obese rats. These findings could prove the mechanistic insightful of *C5* as an option in the treatment of dyslipidemia and the prevention of insulin resistance, hepatic steatosis, and potential risk for diabetes.

## 2. Results

### 2.1. C5 Improved the General Characteristics and Biochemical Parameters of HFD-Induced Obese Rats

High-fat diet-fed vehicle (HFV) rats demonstrated significant increases in calorie intake, body weight (BW), BW gain, liver weight (LW), liver index, and visceral fat weight compared with normal diet-fed rats (NDV) (Table 1). On the other hand, the BW and BW gain in the *C5*-3 and LO groups were normalized similarly to those in the NDV group. *C5* at 3 and 10 mg/kg was also able to restore the liver index to similar levels as those in the NDV group. All the treatments significantly reduced the visceral fat weight when compared with HFV rats (Table 1). A marked increase in the plasma total cholesterol, triglycerides, LDL, insulin, and the homeostasis assessment of insulin resistance index (HOMA index); a decrease in HDL; and impaired kidney and liver function were shown in HFV rats (Table 2). These parameters were reversed by all treatments. *C5* at maximum concentration did not change any parameters in the control rats (ND+*C5*-10) (Table 2).

### 2.2. C5 Improved Hepatic Histological Features and Exhibited Anti-Hepatic Steatosis

**Figure 2:**
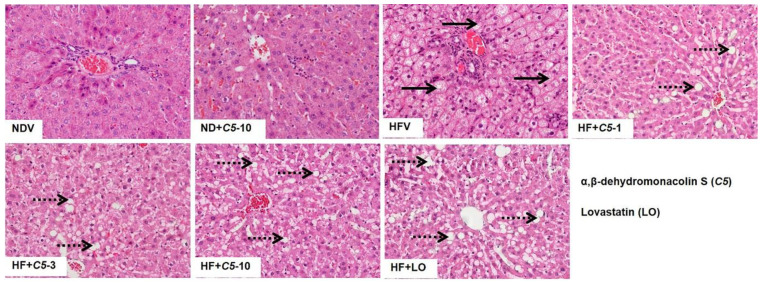
Micrographs of rat liver morphology. Liver tissues from each animal was removed, fixed, embedded, cut, stained by hematoxylin and eosin (H&E). The data were analyzed using bright-field microscopy with original magnification 100× and performed at least 3 times from separate sets of animals. Black arrow indicates microvesicular steatosis and black dot arrow indicates macrovesicular steatosis.

**Figure 3:**
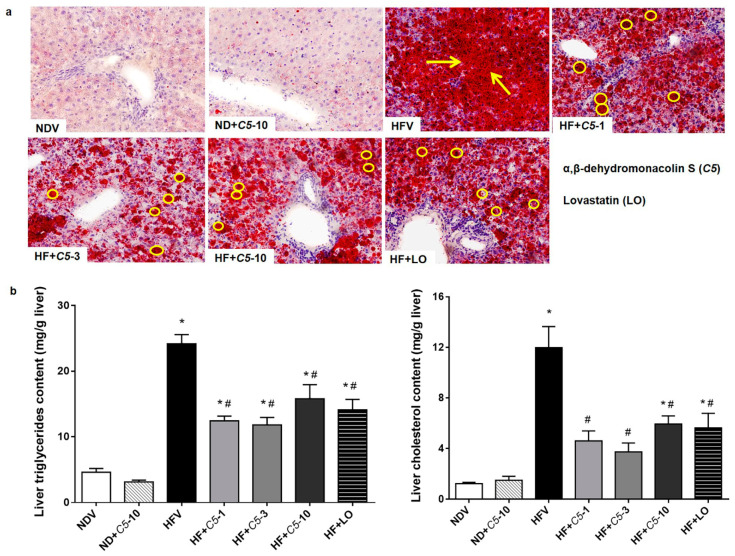
Effect of α,β-dehydromonacolin S (*C5*) on hepatic lipid accumulation. (**a**) Oil-red-o stained liver sections from each experimental group were analyzed using bright-field microscopy (original magnification 100×). The data were repeated at least 3 times from separate sets of animals. The arrow and circle in the panels indicates fat droplet accumulation in liver tissues. (**b**) Triglyceride (TG) and total cholesterol (TC) extracted from liver tissues were quantitated using enzymatic colorimetric assay. Values are mean ± S.E.M (*n* = 6), * *p* < 0.05 represents the significant difference compared with the NDV group and ^#^ *p* < 0.05 represents the significant difference compared with the HFV group.

To determine the effect of *C5* on the liver morphology, hematoxylin and eosin (H&E) staining was performed. The liver morphology in NDV-treated rats was normal, as indicated by the normal shapes of the hepatocytes, a low level of fat accumulation, and the absence of hepatic ballooning, as with the ND+*C5*-treated animals (Figure 2). On the other hand, HFV rat livers demonstrated numerous lipid vacuoles in a pattern of microvesicular steatosis (black arrows) in contrast to NDV. This defect was recovered by administration of both *C5* and LO as shown by a reduction in the number of lipid vacuoles and the alleviation of macrovesicular steatosis (dot arrows) when compared with HFV. In addition, liver sections were stained with oil-red-o (ORO) to identify hepatic steatosis (Figure 3a). The NDV group showed few ORO-positive areas (ORO+), similarly to the ND+*C5*-10 group. In contrast, the HFV group demonstrated a marked increase in neutral lipids, as indi-cated by ORO+ (arrows), compared with NDV. The condensed, red-stained vacuoles were decreased in all treatment groups compared with the HFV group. Correspondingly, the hepatic triglyceride and total cholesterol contents were significantly increased in the HFV group when compared with the NDV group. Again, all treatments had significantly lower lipid contents; liver triglyceride and cholesterol, when compared with the HFV group. There was no difference in the hepatic content in the ND+*C5*-10 group compared with the NDV group (Figure 3b).

### 2.3. C5 Inhibited HMG-CoA Reductase Activity and Expression and Modulated the Lipid Metabolic Gene Expression

**Figure 4:**
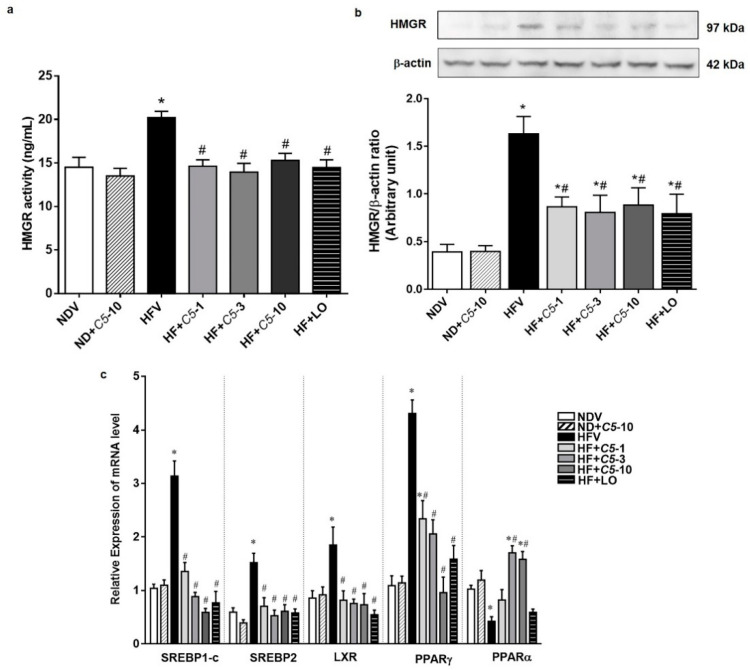
Effect of α,β-dehydromonacolin S (*C5*) on lipid metabolism. (**a**) HMGR activity was determined from liver tissue using ELISA kit. Values are mean ± S.E.M (*n* = 6), (**b**) HMGR protein expression extracted from rat liver tissue was analyzed by Western blotting. A representative blot of HMGR protein expression is shown on the top panel and quantification of relative protein expression in each experimental group presented on the bottom. Anti-β-actin antibody was used as loading control. Values are mean ± S.E.M (*n* = 5), and (**c**) mRNA expression of SREBP-1c, LXRα, PPARγ, SREBP2 and PPARα from each experimental group were compared using qPCR. Each data is expressed as mean ± S.E.M (*n* = 5). * *p* < 0.05 represents the significant difference compared with the NDV group and ^#^ *p* < 0.05 represents the significant difference compared with the HFV group.

To determine the direct effect of *C5*, the HMGR activity and protein expression in liver tissues were investigated in each experimental group. The HMGR activity and protein expression did not differ between the NDV and ND+*C5*-10 groups. However, the HFV group had both increased HMGR activity and increased protein expression when compared with NDV. Consistently, all treatment groups decreased in these parameters when compared with HFV (Figure 4a,b). The HFV group demonstrated increased expression of lipogenic genes, including sterol regulatory element binding pro-tein-1c (SREBP1-c), liver X receptor alpha (LXRα), peroxisome proliferator-activated receptor gamma (PPARγ), and sterol regulatory element-binding protein 2 (SREBP2), as well as decreased peroxisome proliferator-activated receptor alpha (PPARα) mRNA expression, which plays a role in lipolysis, when compared with NDV. In contrast, treatments with both *C5* and LO normalized the expression of these genes. There was no significant difference in the expression of any genes in the ND+*C5*-10 group when compared with the NDV group (Figure 4c).

### 2.4. C5 Modulated Hepatic Lipid Transporter Gene and Protein Expression 

**Figure 5:**
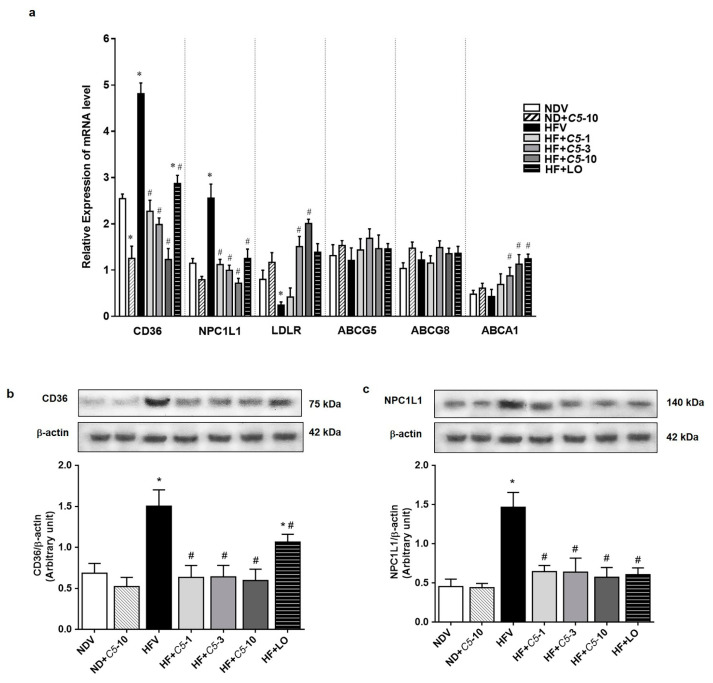
α,β-dehydromonacolin S (*C5*) modulates lipid transporter gene and protein expression. (**a**) The mRNA expression of lipid transporters including CD36, NPC1L1, LDLR, ABCG5/8 and ABCA1 were quantitatively determined using qPCR. (**b**) CD36 and (**c**) NPC1L1 protein expression extracted from rat liver tissue were analyzed by Western blotting. A representative blot of CD36 and NPC1L1 protein expression is shown on the top panel and quantification of relative protein expression in each experimental group presented on the bottom. Anti-β-actin antibody was used as loading control. Values are mean ± S.E.M (*n* = 5). * *p* < 0.05 represents the significant difference compared with the NDV group and ^#^ *p* < 0.05 represents the significant difference compared with the HFV group.

As shown in Figure 5a, there were no significant differences in the sterol efflux transporters, ATP-binding cassette subfamily G, members 5 and 8 (ABCG5/8), mRNA expression among the experimental groups. *C5* at 3 and 10 mg/kg demonstrated significantly increased ATP-binding cassette subfamily A, member 1 (ABCA1) mediates the efflux of excess cholesterol and nascent HDL particles similarly to the effect of LO without any changes in the other groups (Figure 5a). HFV rat liver presented markedly increased influx lipid transporters, including cluster of differentiation 36 (CD36) and Niemann-Pick C1 Like 1 (NPC1L1), and decreased LDLR mRNA expression when compared with NDV. The administration of *C5* and LO significantly decreased both CD36 and NPC1L1 while increasing the LDLR mRNA expression when compared with HFV (Figure 5a). Consistent with mRNA expression, *C5* decreased the protein expression of both hepatic lipid transporters CD36 and NPC1L1 when compared with HFV, similarly to LO (Figure 5b,c).

### 2.5. Antioxidant and Anti-Inflammatory Effects of C5 

**Figure 6:**
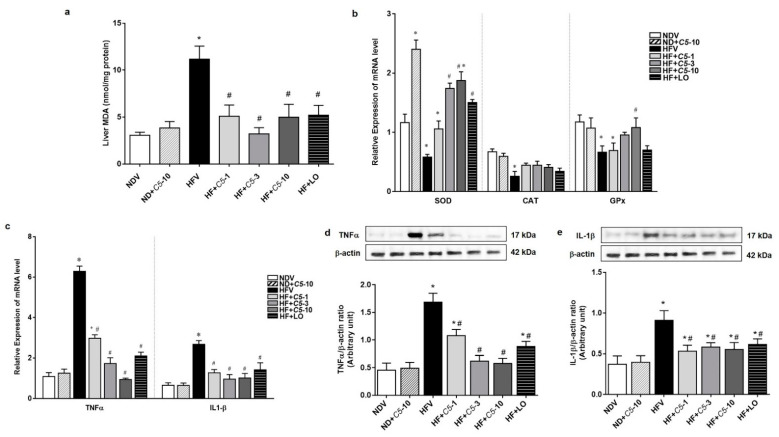
Antioxidant and Anti-inflammatory effects of α,β-dehydromonacolin S (*C5*). (**a**) The malondialdehyde (MDA) level (*n* = 6), (**b**) mRNA expression of Cu-ZnSOD, CAT, and GPx, (**c**) TNF-α and IL1-β mRNA expression, (**d**) expression of TNF-α, and (**e**) IL1-β protein extracted from rat liver tissues. A representative blot of TNF-α, and IL1-β protein expression is shown on the top panel and quantification of relative protein expression in each experimental group presented on the bottom. Anti-β-actin antibody was used as loading control. Values are shown as mean ± S.E.M (*n* = 5), * *p* < 0.05 represents the significant difference compared with the NDV group and ^#^ *p* < 0.05 represents the significant difference compared with the HFV group.

To further determine the effect of *C5* on hepatic oxidative stress, lipid peroxidation as indicated by the level of MDA and expression of antioxidant genes, including catalase (CAT), glutathione peroxidase (GPx), and Cu-Zn superoxide dismutase (Cu-ZnSOD) were determined. There was no significant difference in MDA, CAT, and GPx between the NDV and ND+*C5*-10 groups (Figure 6a,b). The HFV group presented significantly increased liver MDA and decreased antioxidant gene expression compared with the NDV group. In contrast, all treatments significantly decreased the liver MDA levels and normalized antioxidant gene transcripts when compared with the HFV group. This latter effect was predominantly seen in the Cu-ZnSOD gene expression in the ND+*C5*-10 group when compared with NDV (Figure 6a,b). Consistent with oxidative stress, HFV rat liver tissues highly expressed tumor necrosis factor alpha (TNF-α) and interleukin 1 beta (IL1-β) mRNA and protein expression when compared with the NDV group. Treatments with *C5* and LO decreased both the mRNA and protein expression profiles of TNF-α and IL1-β (Figure 6c–e).

### 2.6. C5 Suppressed Hepatic Gluconeogenesis and Improved Insulin Signaling

**Figure 7:**
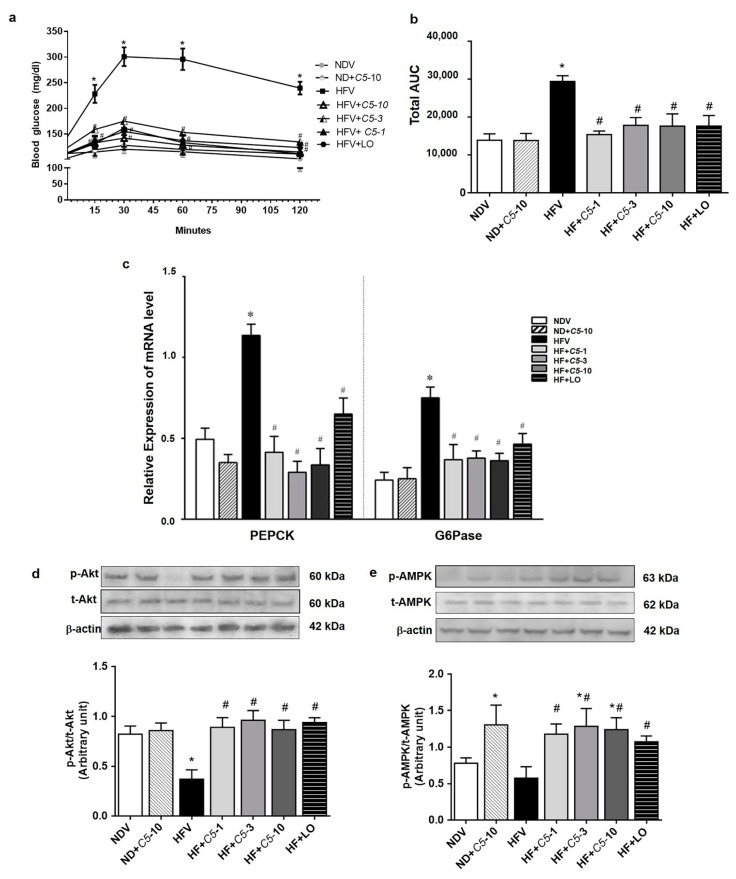
Effect of α,β-dehydromonacolin S (*C5*) against hepatic glucose overproduction. (**a**) Blood glucose level was measured at 0, 15, 30, 60 and 120 min after injecting with alanine (100 mg/kg BW) (*n* = 6), (**b**) total area under the curve from (A) was analyzed. (**c**) mRNA expression of PEPCK and G6Pase, (**d**) protein expression of *p*-AMPK and total AMPK protein, and (**e**) *p*-Akt and total Akt protein expression extracted from rat liver tissues. A representative blot of *p*-AMPK, total AMPK, *p*-Akt, and total Akt protein expression is shown on the top panel and quantification of relative protein expression in each experimental group presented on the bottom. Anti-β-actin antibody was used as loading control. Values are shown as mean ± S.E.M (*n* = 5), * *p* < 0.05 represents the significant difference compared with the NDV group and ^#^ *p* < 0.05 represents the significant difference compared with the HFV group.

The blood glucose level after alanine loading at 15, 30, 60, and 120 min in HFV was markedly increased when compared with NDV, however, this was restored by both *C5* and LO treatments (Figure 7a,b). Consistent with the gluconeogenesis assay, phosphoenolpyruvate carboxykinase (PEPCK) and glucose-6-phosphatase (G6Pase) transcripts in the HFV group significantly increased while these genes were down-regulated after both *C5* and LO treatments (Figure 7c). Similarly, a marked decrease in the expression ratio of *p*-Akt/Akt was shown in HFV while the *p*-AMPK/AMPK ratio tended to decrease. On the other hand, both *C5* and LO improved the phosphorylation or activity of these two proteins (Figure 7d,e). ND+*C5*-10 had a significantly increased *p*-AMPK/AMPK expression ratio when compared with the NDV group (Figure 7e).

## 3. Discussion

This study demonstrates the lipid-lowering effects with high efficacy of α,β-dehydromonacolin S, *C5*, in HFD-induced hepatic steatosis in rats. The mechanisms by which *C5* reduced the lipid contents in both plasma and hepatocytes are clarified and proposed to involve direct binding to HMGR, the rate-limiting enzyme of de novo cholesterol synthesis. The modulation of genes and proteins involved in hepatic glucose and lipid metabolism and transporters, and the glucose metabolism contribute to suppression of hepatic gluconeogenesis and improved insulin resistance. 

Our previous study demonstrated that the inactive lactone of *C5* directly binds to the active site of HMGR at the catalytic domain with low affinity (IC_50_ value of 387 ± 70 μM) compared to readily active pravastatin with an IC_50_ value of 0.4 ± 0.1 μM in an in vitro model. The potency of *C5* is slightly higher than that of lovastatin but much lower than that of pravastatin, which inhibited HMGR activity by ∼90.4% [19]. To support this finding, the present study demonstrates that such *C5* action had a higher potency than that of lovastatin, as indicated by the lower effective dose of *C5* at 1 and 3 mg/kg required to produce lipid-lowering effects. In addition to *C5*, several other compounds extracted from fungi have been identified to exhibit lipid-lowering actions. For instance, the purification of metabolite extracted from *Fusarium solani* FG319 showed an anti-atherosclerotic effect by inhibiting HMGR activity and exerting lipid-lowering action in vitro [20]. The hexane fraction isolated from *Hericium erinaceus* was also found to be a potent inhibitor for reducing both LDL and HMGR activity in vitro [21]. Recently, a methanolic extract of Ficus virens bark (FVBM) showed an inhibitory effect on HMGR activity paralleled with hypolipidemic activity in triton WR-1339-induced hyperlipidemic rats. In addition, the lipid-lowering action of FVBM was equivalent in efficacy to that of atorvastatin, a lipophilic statin [22]. Although the efficacy of anti-lipidemia of *C5* validated in our study was found to be superior than that of lovastatin, there are insufficient pieces of knowledge that require extensive research in order to build a comprehensive clinical implication of *C5*. This includes: anti-atherosclerotic effects of *C5* for prevention of CVD; the major muscle adverse side effect; and chronic toxicity studies of *C5* and its safety prior to initiation of the various clinical trials in humans. A previous study found that a high-fat diet up-regulated the site-1 protease (S1P) gene, a cleavage enzyme for activating SREBP-2 leading to increased hepatic HMGR expression and activity in mice [4]. This evidence is also seen and strongly associated with obese, hepatic steatosis, and diabetic patients [5]. Consequently, excessive levels of hepatic cholesterol and its oxysterol metabolites activate LXRα and additively induce the SREBP-1c transcript, exacerbating hepatic lipogenesis and steatosis [6,23]. In this study, we found that *C5* could reduce these regulatory factors including SREBP-2, HMGR, LXRα, and SREBP-1c, for controlling hepatic cholesterol and triglyceride contents in HFD condition. Thus, not only does *C5* exhibit lipid lowering effects, but it also shows anti-hepatic steatosis effects, and potentially prevent the development of NAFLD and NASH, of which there is no approved therapeutic agent. NAFLD is the excessive hepatic lipid accumulation strongly associated with insulin resistance, diabetes mellitus, and metabolic syndrome [24]. A low dose of simvastatin (4–10 µM) prevented NAFLD in oleic acid-induced NAFLD in HepG2 cells [25]. In addition, 20 mg of atorvastatin daily combined with vitamins C and E effectively reduced hepatic steatosis by 71% in NAFLD patients after 4 years of active therapy [26]. Like statins, *C5* dramatically improved hepatic steatosis as shown by restoring the liver morphology and decreasing the hepatic lipid content in HFD-induced hepatic steatosis rats. Therefore, *C5* might be an optional candidate for treating dyslipidemia and hepatic steatosis. 

The molecular mechanisms by which statin lowers cholesterol have been studied extensively. For instance, the administration of atorvastatin in hypercholesterolemic patients reduced SREBP-1c mRNA expression, resulting in improved serum lipid profiles [27]. Similarly, this study shows that *C5* not only decreased SREBP-1c, but also SREBP-2 mRNA expression, leading to the inhibition of down-stream targeting of the latter, HMGR, and cholesterol synthesis, which, in turn, reduced triglyceride accumulation and improves hepatic steatosis. *C5* also dominantly activated AMPK, which is the upstream kinase that directly phosphorylates SREBP-1c and SREBP-2. To support this notion, inhibition of these SREBP transcription factors led to the suppression of hepatic lipogenesis in mice [28]. In addition, previous study showed that 5-aminoimidazole-4-carboxamide ribonucleotide (AICAR) activated AMPK, which subsequently phosphorylates SREBP-2 in mouse livers, resulting in the amelioration of hepatic steatosis and hypercholesterolemia [29], while irisin improved hepatic steatosis via the sequential activation of AMPK and inhibition of SREBP-2 in high-fat diet-fed mice [30]. Atorvastatin increased hepatic PPARα mRNA and protein similarly to the effect of fenofibrate, leading to improved lipid metabolic phenotypes in senescent rats [31]. The FFA transporter, CD36, expressed in macrophages was down-regulated, leading to a reduction of lipids and inflammation in patients suffering from acute coronary syndromes [32]. Like atorvastatin, lipophilic pitavastatin prevents the uptake of oxidized-LDL via the down-regulation of CD36 in human phagocytic cells through PPARγ [33]. These lines of evidence were consistent with the dose-dependent effects of *C5* on down-regulating PPARα- and PPARγ-mediated CD36 function. Simultaneously, *C5* down-regulated NPC1L1, the target protein of the lipid-lowering drug ezetimibe, through LXRα activation and increased LDLR gene expression. Recently, naringin was shown to activate AMPK, resulting in down-regulation of the SREBP2 and proprotein convertase subtilisin/kexin type 9 (PCSK9), which, in turn, up-regulated LDLR and reduced the plasma and hepatic lipids in obese C57BL/6J mice [34]. More recent study suggested that the combination of statins with either ezetimibe or PCSK9 monoclonal antibody exerts efficient lipid-lowering action while combined with three types of lipid-lowering drugs, which requires further studies [35]. Hence, *C5* appears to be an option with robust lipid-lowering effects via the modulation of the lipid metabolism and hepatic lipid transporters.

A recent study showed that prolonged inflammation could exacerbate liver tissue injury, causing progressive development of NAFLD, nonalcoholic steatohepatitis (NASH) and liver fibrosis in NASH patients [36]. Rosuvastatin attenuated pro-inflammatory cytokines, preventing cardiovascular disease and improving clinical outcomes [37]. Furthermore, atorvastatin reduces the pro-inflammatory markers TNFα, IL-1β, and IL-6 in hypercholesterolemic patients [38], while simvastatin also reduced the expression of IL-6, IL-8, and monocyte chemoattractant protein-1 (MCP1) in peripheral blood mononuclear cells in hypercholesterolemic patients [39]. Atorvastatin also improved the endothelial function mediated by AMPK activation in both in vitro and in vivo models [40]. The phosphorylation of AMPK and Akt by metformin also deactivated the gluconeogenic enzymes PEPCK and G6Pase, thereby, decreasing hepatic glucose production [41]. In agreement with the pleiotropic effects of statins, this study revealed that *C5* not only had a lipid-lowering action but also exhibited a hepatoprotective effect by reducing oxidative stress and inflammation and preventing the consequences of hyperglycemia and potential risk of diabetes mellitus. The direct effect of AMPK activation by *C5* as seen in the control rats in this study was similar to that of the direct action of metformin. Therefore, due to its high efficacy and safety characteristics, *C5* has the potential to be developed as a treatment for dyslipidemia, hepatic steatosis, and to prevent NOD. However, whether *C5* acts as an AMPK activator requires further investigation.

At present, the production of novel chemically synthesized statins is inspired by the structures of naturally occurring statin molecules. However, high efficacy lipid-lowering action together with a critical role for preventing cardiovascular disease (CVD) are still needed. The negative effects of statins associated with increase insulin resistance, hepatic gluconeogenesis, and the risk of NOD are recently disclosed in several models including in vitro, in vivo, as well as observational studies, clinical trials, and meta-analyses [11,12,13]. Although the precise molecular mechanisms of statins that induced NOD are unclear, persistent activation of gluconeogenesis is documented to contribute to T2DM [13]. Unlike adverse effects of statins-induced T2DM, 6-weeks treatment with *C5* neither induce insulin resistance, impair hepatic gluconeogenesis, nor produce hepatoxicity. Ultimately, *C5* exerts beneficial effects in the liver as indicated by, first, decreasing the gluconeogenic genes PEPCK and G6Pase, and second, increasing AMPK/Akt activation, which is a key control in both glucose and lipid metabolism. Hence, *C5* may be a novel option for treating dyslipidemia and hepatic steatosis, and reducing the risk of diabetes mellitus with which could be further develop into drug discovery and development.

## 4. Methods

### 4.1. Chemicals

Monoclonal rabbit anti-HMGR and monoclonal mouse anti-β-actin were obtained from Abcam (Cambridge, MA, USA). Polyclonal rabbit anti-Niemann–Pick C1-Like 1 (NPC1L1) and cluster of differentiation 36 (CD36) were purchased from Novus biological (CO, USA). Polyclonal rabbit anti-AMP-activated protein kinase (AMPK), phosphorylated Thr172 AMPK (*p*-AMPK), and complete protease inhibitor cocktail were purchased from Merck (Darmstadt, Germany). Polyclonal rabbit anti-Akt and phosphorylated Akt (*p*-Akt, Ser473) were obtained from Cell Signaling Technology (Danvers, MA, USA). Polyclonal rabbit anti-TNF-α and monoclonal anti-IL1β were obtained from EMD Millipore (Burlington, CA, USA). CelLytic™ MT cell lysis reagent, cholesterol and DL-methionine were obtained from Sigma-Aldrich (St. Louis, MO, USA). Casein was prepared from pasteurized milk (Dairy farming promotion organization of Thailand, Chiang Mai, Thailand). Vitamin and mineral mixture was purchased from Namping Pasusad (2000) Co. Ltd. (Chiang Mai, Thailand). Lard and yeast powder were obtained from commercial sources. All other chemicals used were of high purity.

### 4.2. Preparation of Lovastatin and α,β-Dehydromonacolin S 

Lovastatin (LO) and α,β-dehydromonacolin S (*C5*) [19] were produced by the soil-derived fungus Aspergillus sclerotiorum PSU-RSPG178 isolated from a soil sample collected from the Plant Genetic Conservation Project under the Royal Initiation of Her Royal Highness Princess Maha Chakri Sirindhorn at Ratchaprapa Dam in Suratthani Province, Thailand. The structures (Figure 1) were validated using our recently reported spectroscopic techniques [19].

### 4.3. Animal Induction

Forty-two male Wistar rats were obtained from Nomura Siam International (Bangkok, Thailand). The animal facilities and protocols were approved by the Laboratory Animal Care and Use Committee at the Faculty of Medicine, Chiang Mai University, Chiang Mai, Thailand (Protocol number: 14/2561). All rats were housed in a room maintained at 25 ± 1 °C on a 12:12 h dark–light cycle and acclimated for 1 week with free access to chow and water. The rats were divided randomly into two groups. The first group received a normal diet (ND) (C.P. Mice Feed Food no. 082, Bangkok, Thailand) containing 19.77% of fat from the total energy in the diet (%E) (*n* = 12). The second group received a high-fat diet (HFD) containing approximately 59.28% energy from fat, including lard, ad libitum (*n* = 30). The composition of HFD, modified from a previous study [42], is shown in Table S1. After the 12th week, the animals in each group were divided randomly and equally into one of the following treatments (*n* = 6); ND fed vehicle, 1% carboxymethyl cellulose, designated as NDV, ND treated with *C5* at 10 mg/kg (ND+*C5*-10), high-fat (HF) fed vehicle (HFV), HF treated with *C5* at 1, 3, or 10 mg/kg (HF+*C5*-1, HF+*C5*-3, and HF+*C5*-10), and HF treated with lovastatin at 10 mg/kg (HF+LO). The animals were administered vehicle, *C5*, or LO by gavage feeding daily for another 6 weeks. The body weight, food intake, and water intake were recorded daily. For the alanine tolerance test (ATT), 18 h-fasted animals were injected intraperitoneally with L-alanine at 100 mg/kg. Blood samples were collected at 0, 15, 30, 60, 90, and 120 min, and the blood glucose level at each time point was determined using an enzymatic assay kit obtained from Erba Lachema (Brno, Czech Republic). The data were calculated as the total area under the curve (total AUC). After sacrifice, blood and organ tissues were collected and stored at −80 °C for further investigations.

### 4.4. Determination of Plasma Metabolic Parameters

The quantitative total plasma glucose, triglycerides, cholesterol, and high-density lipoprotein (HDL) were determined using an enzymatic assay kit obtained from Biotech Co., Ltd. (Bangkok, Thailand), and the LDL was subsequently calculated. The aspartate aminotransferase (AST) and alanine aminotransferase (ALT) were determined using a colorimetric assay kit at the Small Animal Teaching Hospital, Faculty of Veterinary Medicine, Chiang Mai University. Plasma insulin concentrations were determined using a sandwich ELISA assay kit from LINCO Research (Merck, Darmstadt, Germany). HOMA index was then calculated to estimate the insulin resistance condition by the following formula:

$$\frac{\mathrm{fasting}\;\mathrm{plasma}\;\mathrm{insulin}\;\left(µU/\mathrm{mL}\right)\times \;\mathrm{fasting}\;\mathrm{plasma}\;\mathrm{glucose}\;\left(\mathrm{mmol}/L\right)}{22.5}$$

### 4.5. Determination of the Liver Morphology, Lipid Accumulation, and Lipid Content

The liver histology, lipid accumulation analysis, and lipid content quantification were performed according to our previous study [43]. Briefly, 4% paraformaldehyde-fixed rat liver sections were stained with hematoxylin and eosin (H&E) to evaluate the liver morphology. A microscopic examination was carried out, and photographs were taken under a regular light microscope. For lipid accumulation, 10-µm liver cryo-sections were dehydrated in 70% alcohol and stained with 2% ORO for 20 min. The sections were counterstained with hematoxylin, and the neutral lipid content was subsequently determined using a bright-field microscope. The liver and fecal cholesterol and triglyceride contents were determined by extracting liver tissues and dried feces with chloroform and isopropanol at a ratio of 7:11, and the levels of the lipids were subsequently determined using an enzymatic colorimetric assay as mentioned above.

### 4.6. Determination of Hepatic HMGR Activity and Lipid Peroxidation 

Liver tissue lysate was prepared by homogenizing in ice-cold phosphate buffer saline (PBS), and the HMGR activity was subsequently determined according to the manufacturer’s protocol using an ELISA kit from Cloud-Clone Corp (Houston, TX, USA). The levels of malondialdehyde (MDA), which represents lipid peroxidation in rat liver tis-sue lysates, were measured using the commercial thiobarbituric acid assay from Cayman Chemical (Ann Arbor, MI, USA).

### 4.7. Expression of Genes Involved Hepatic Lipid Metabolism, Lipid Transporters, Antioxidant, and In-Flammation Using the Quantitative Polymerase Chain Reaction (qPCR) Technique 

The total RNA from freshly isolated rat liver tissues was purified using TRIzol reagent (Thermo Fisher Scientific, Waltham, MA, USA), according to the manufacturer’s instructions. First-strand cDNA was subsequently generated using the iScript cDNA synthesis kit (Macrogen, Geumcheon-gu, Seoul, Korea), and qPCR was performed using SYBR green real-time PCR master mix (Bioline, London, UK) on an ABI 7500 (Life Technologies, New York, NY, USA). The forward and reverse primers were taken from previous studies and recent studies of ours [44,45,46] or designed and purchased from Macrogen (Geumcheon-gu, Seoul, Korea), as shown in Table S2.

### 4.8. Expression of Protein Involved in Hepatic Lipid Transporters and Insulin Signaling Using Western Blotting Analysis

Liver tissue lysate was prepared by homogenizing liver tissue in CelLytic™ MT cell lysis reagent containing protease inhibitors (Merck, Darmstadt, Germany). The lysate was centrifuged at 5000× *g* for 10 min at 4 °C, and the supernatant was designated as the whole cell lysate. The protein concentration in each sample was determined using the Bradford assay (Bio-Rad, Hercules, CA, USA), and the samples were stored at −80 °C prior to use. For electrophoresis, samples were resolved in 4X Laemmli buffer, electrophoresed in 10% SDS-PAGE, and transferred onto polyvinylidene difluoride membranes (GE Healthcare, Chicago, IL, USA). The membrane was incubated with either polyclonal anti-rabbit or anti-mouse antibodies (see details in Figure legends) overnight. The membrane was then washed with tris-buffer saline with 0.1% triton x-100 (TBS-T) and incubated with horseradish peroxidase-conjugated secondary goat anti-rabbit or anti-mouse IgG (Merck, Darmstadt, Germany) for 1 h. The target protein was detected using Super Signal West Pico chemiluminescent substrate (GE Healthcare, WI, USA) and quantitatively analyzed using the Image J program from the Research Services Branch of the National Institute of Health/National Institute of Mental Health (NIH/NIMH) (Bethesda, MD, USA).

### 4.9. Statistical Analysis

The data are expressed as the mean ± S.E.M. Statistical differences were assessed using one-way ANO-VA followed by the LSD post hoc test. Statistical analyses were conducted using Statistical Product and Service Solutions (SPSS) Statistical Software version 23 (IBM Corp., New York, NY, USA). Differences were considered significant when *p* < 0.05. 

## 5. Conclusions

This study suggests that *C5* isolated from the soil-derived fungus, Aspergillus sclerotiorum PSU-RSPG178, has a high efficacy regarding lipid-lowering effects and improved glucose metabolism, which could protect the progression of NAFLD and reduce the risk for diabetes mellitus in high-fat diet-induced obese rats. *C5* directly inhibited HMGR activity and expression, resulting in a reduction of cholesterol levels. Decreased cholesterol leads to improved hepatic steatosis by inducing lipogenesis and stimulating lipolytic factors. *C5* could prevent the risk for diabetes mellitus by decreasing hepatic gluconeogenesis together with antioxidant, anti-inflammatory, and anti-insulin resistant actions mediated through AMPK/Akt. Collectively, α,β-dehydromonacolin S, *C5*, has a high potential for development as an option for treating dyslipidemia, and preventing the risk of NAFLD, diabetes mellitus, and metabolic syndrome.

## Figures and Tables

**Table 1:** Effect of α,β-dehydromonacolin S (*C5*) on daily intake, body weight, and organ weights in normal and high-fat diet-induced obese rats.

Parameters	NDV	ND+*C5*-10	HFV	HF+*C5*-1	HF+*C5*-3	HF+*C5*-10	HF+LO
Food intake	30.46 ± 1.87	28.46 ± 2.18	29.73 ± 1.86	28.76 ± 1.46	27.65 ± 2.25	30.12 ± 2.83	29.75 ± 2.14
Calories intake (kcal/day)	122.92 ± 1.45	115.27 ± 2.45	159.05 ± 1.75 *	146.39 ± 2.17 *	147.92 ± 1.45 *	161.142 ± 2.13 *	159.16 ± 1.97 *
Water intake (ml/day)	20.84 ± 3.15	20.12 ± 1.56	20.46 ± 2.46	19.65 ± 2.86	20.46 ± 2.73	21.78 ± 3.45	20.56 ± 2.16
BW (g)	546.43 ± 7.31	510.00 ± 9.91	637.50 ± 20.24 *	633.57 ± 26.90 *	610.00 ± 16.22	648.57 ± 37.30 *	615.00 ± 24.86
BW gain (g)	371.43 ± 5.61	340.83 ± 10.60	467.50 ± 19.86 *	457.86 ± 25.89 *	436.43 ± 15.65	475.00 ± 37.64 *	441.43 ± 25.21
LW (g)	13.07 ± 0.62	14.34 ± 0.59	29.48 ± 2.61 *	23.78 ± 2.44	21.14 ± 1.32	24.63 ± 2.43	22.51 ± 2.19
Liver index *	2.40 ± 0.12	2.81 ± 0.10	4.72 ± 0.58 *	3.81 ± 0.45 *	3.49 ± 0.27	3.56 ± 0.22	3.68 ± 0.37 *
Visceral fat weight (g)	25.66 ± 1.32	27.61 ± 3.86	65.32 ± 2.26 *	43.48 ± 3.38 *^#^	30.64 ± 1.18 ^#^	32.89 ± 1.48 ^#^	41.15 ± 3.29 *^#^

Values shown are mean ± S.E.M (*n* = 6), * *p* < 0.05 represents the significant difference compared with the NDV group; ^#^ *p* < 0.05 represents the significant difference compared with the HFV group. * Liver index was calculated by liver weight divided by body weight and multiply by 100.

**Table 2:** Effect of α,β-dehydromonacolin S (*C5*) on blood biochemical parameters in normal and high-fat diet-induced obese rats.

Plasma Parameters	NDV	ND+*C5*-10	HFV	HF+*C5*-1	HF+*C5*-3	HF+*C5*-10	HF+LO
Cholesterol (mg/dL)	73.26 ± 3.21	73.48 ± 4.60	115.10 ± 8.41 *	83.43 ± 4.18 ^#^	70.57 ± 3.60 ^#^	86.22 ± 4.12 ^#^	79.39 ± 3.19 ^#^
Triglyceride (mg/dL)	43.86 ± 2.94	51.04 ± 3.10	80.29 ± 4.40 *	60.69 ± 5.61 ^#^	55.54 ± 3.09 ^#^	64.39 ± 5.17 ^#^	58.72 ± 6.67 ^#^
HDL (mg/dL)	31.70 ± 2.99	28.47 ± 1.89	14.69 ± 1.05 *	26.26 ± 1.90 ^#^	28.71 ± 2.02 ^#^	31.36 ± 2.00 ^#^	30.39 ± 1.68 ^#^
LDL (mg/dL)	32.78 ± 5.42	34.80 ± 5.33	84.35 ± 8.42 *	45.03 ± 4.78 ^#^	30.76 ± 5.39 ^#^	41.99 ± 6.11 ^#^	36.71 ± 2.84 ^#^
Glucose (mg/dL)	117.48 ± 9.21	117.96 ± 15.77	137.75 ± 13.73	116.41 ± 10.32	120.88 ± 10.35	122.69 ± 9.63	124.27 ± 12.01
Insulin (ng/mL)	1.60 ± 0.41	1.51 ± 0.12	3.94 ± 0.37 *	1.47 ± 0.29 ^#^	1.95 ± 0.35 ^#^	1.86 ± 0.30 ^#^	1.63 ± 0.27 ^#^
HOMA index	11.01 ± 2.12	10.89 ± 2.13	32.41 ± 3.62 *	11.41 ± 3.07 ^#^	14.26 ± 3.35 ^#^	14.05 ± 2.57 ^#^	11.55 ± 1.62 ^#^
AST (U/L)	153.71 ± 13.42	142.67 ± 16.79	222.00 ± 21.32 *	123.29 ± 13.51 ^#^	133 ± 9.88 ^#^	166.43 ± 11.90	128.14 ± 7.22 ^#^
ALT (U/L)	86.57 ± 15.53	79.83 ± 15.06	253.00 ± 15.62 *	66.43 ± 9.12 ^#^	70.57 ± 13.71 ^#^	79.29 ± 10.63 ^#^	81.14 ± 9.36 ^#^
BUN (mg/dL)	21.18 ± 2.50	23.71 ± 1.51	43.83 ± 13.04 *	23.64 ± 2.00 ^#^	23.03 ± 2.35 ^#^	27.60 ± 1.85 ^#^	26.24 ± 2.77 ^#^
Creatinine (mg/dL)	0.81 ± 0.03	0.80 ± 10.03	1.39 ± 10.07 *	0.80 ± 0.06 ^#^	0.92 ± 0.03 ^#^	1.12 ± 0.05 *^#^	0.97 ± 0.07 ^#^
GFR (ml/min)	2.36 ± 0.19	2.10 ± 10.24	3.39 ± 10.23 *	2.31 ± 0.19	2.42 ± 0.25	2.54 ± 0.26	2.68 ± 0.20

HDL, high-density lipoprotein cholesterol, LDL, low-density lipoprotein cholesterol, HOMA index; homeostasis assessment of insulin resistance index. Each value is expressed as mean ± S.E.M (*n* = 6). * *p* < 0.05 indicates the significant differences from the NDV group and ^#^ *p* < 0.05 indicates the significant differences from the HFV group.

## Data Availability

The data presented in this study are available on request from the corresponding author.

## Supplementary Materials

The following are available online at https://www.mdpi.com/article/10.3390/ph14040375/s1, Table S1: Composition of high-fat diet. Table S2: Primer sequences and expected amplicon sizes for gene amplification.

## Abbreviations

ABCA1: ATP-binding cassette, subfamily A, member 1; ABCG5/8, ATP-binding cassette, subfamily G, member 5/8; AMPK, AMP-dependent kinase; Akt, serine/threonine protein kinase B; CAT, catalase; CD36, cluster of differentiation 36; Cu-Zn SOD, copper zinc superoxide dismutase; G6Pase, glucose-6-phosphatase; GPx, glutathione peroxidase; HDL, high-density lipoprotein cholesterol, HMGR, 3-hydroxy-3-methylglutaryl coenzyme A reductase; HOMA, homeostasis assessment of insulin resistance; IL-1β, interleukin 1 beta; LDLR, low-density lipoprotein choles-terol receptor; LXRα, liver X receptor alpha; NPC1L1, Niemann-Pick C1 Like 1; ORO, oil-red-o; PEPCK, phosphoenolpy-ruvate carboxykinase; PPARγ, peroxisome proliferator-activated receptor gamma; PPARα, peroxisome proliferator-activated receptor alpha; SOD, superoxide dismutase; SREBP1-c, sterol regulatory element binding protein-1c; SREBP2, sterol regulatory element-binding protein 2; TNF-α, tumor necrosis factor alpha.
