# Supplementary material for: High-Efficacy α,β-Dehydromonacolin S Improves Hepatic Steatosis and Suppresses Gluconeogenesis Pathway in High-Fat Diet-Induced Obese Rats
Source: Pharmaceuticals (Basel). 2021 Apr 17;14(4):375. doi: 10.3390/ph14040375 (PMC8073358; doi:10.3390/ph14040375)
Supplement: Supplementary file 1 [file pharmaceuticals-14-00375-s001.zip › pharmaceuticals-1134847-supplementary.pdf]

Table S1. Composition of high-fat diet.

Ingredients	Diet *g/kg*
Powdered normal diet*	365
Lard	310
Casein	250
Cholesterol	10
Vitamin and mineral mixture	60
DL-methionine	3
Yeast powder	1
Sodium chloride	1

*Powdered normal diet was from the commercial source (C.P. Mice Feed Food no. 082, Bangkok, Thailand)

Table S2. Primer sequences and expected amplicon sizes for gene amplification.

cDNA	Genbank Acc. no.	Forward primer (5′ to 3’)	Reverse primer (5′ to 3’)	Amplicon size (bp)
CD36	NM031561.2	GACAATCAAAAGGGAAGTTG	CCTCTCTGTTTAACCTTGAT	159
NPC1L1	NM001002025.1	CCACGAGAGGTCCACATTGG	GAAGAAGCAGATGGCCTCAGA	87
LDLR	NM001195800.1	CAGCTCTGTGTGAACCT	TTCTTCAGGTTGGGGATCA	188
ABCG5	NM022436.2	GGGAAGTGTTTGTGAACGGC	GTGTATCTCAGCGTCTCCCG	121
ABCG8	NM130414.2	CGTCAGATTTCCAATGACTTCCG	TCCGTCCTCCAGTTCATAGTACA	241
ABCA1	NM178095.2	ACGAGATTGATGACCGCCTC	AGCATCCACCCCACTCTCTTC	110
PPARα	NM013196.1	AATCCACGAAGCCTACCTGA	GTCTTCTCAGCCATGCACAA	132
PPARγ	NM013124.3	CCCTGGCAAAGCATTTGTAT	GGTGATTTGTCTGTTGTCTTTCC	100
SREBP-1c	NM001276708.1	GGAGCCATGGATTGCACATT	GCTTCCAGAGAGGAGCCCAG	185
SREBP2	NM_001033694.1	CCGGGAAGAAGAGAGCTGTG	CAGACGACATCGGGACCAAG	144
LXRα	NM_031627.2	GCTCTCCGAGATCTGGG	TCCTCAGTCTGCTCCACC	109
Cu-Zn SOD	X05634	GCAGAAGGCAAGCGGTGAAC	TAGCAGGACAGCAGATGAGT	387
GPx	NM030826	CTCTCCGCGGTGGCACAGT	CCACCACCGGGTCGGACATAC	297
CAT	NM012520.2	CCTCCTCGTTCAAGATGTGGTTTTC	CGTGGGTGACCTCAAAGTATCCAAA	122
TNFα	NM012675	CCCAAAGGGAAGAGAAGTTC	CCACTTGGTGGTTTGCTACA	132

IL-1β	NM031512	GTGATGTTCCCATTAGACAGC	CTTTCATCACACAGGACAGG	228
PEPCK	NM_198780.3	CTCACCTCTGGCCAAGATTGGTA	GTTGCAGGCCCAGTTGTTGA	190
G6Pase	NM_013098.2	AACGTCTGTCTGTCCCGGATCTAC	ACCTCTGGAGGCTGGCATTG	133
Actin	NM031144	CCTAAGGCCAACCGTGAAAA	GGAGCGCGTAACCCTCATAG	181
